# Effectiveness of Universal School-Based Screening vs Targeted Screening for Major Depressive Disorder Among Adolescents: A Trial Protocol for the Screening in High Schools to Identify, Evaluate, and Lower Depression (SHIELD) Randomized Clinical Trial

**DOI:** 10.1001/jamanetworkopen.2019.14427

**Published:** 2019-11-01

**Authors:** Deepa L. Sekhar, Krista L. Pattison, Alexandra Confair, Alissa Molinari, Eric W. Schaefer, James G. Waxmonsky, Leslie R. Walker-Harding, Perri Rosen, Jennifer L. Kraschnewski

**Affiliations:** 1 Department of Pediatrics, Penn State College of Medicine, Hershey, Pennsylvania; 2 Department of Public Health Sciences, Penn State College of Medicine, Hershey, Pennsylvania; 3 Department of Psychiatry, Penn State College of Medicine, Hershey, Pennsylvania; 4 Department of Pediatrics, Seattle Children’s, Seattle, Washington; 5 Garrett Lee Smith Youth Suicide Prevention Grant, Harrisburg, Pennsylvania; 6 Department of Medicine, Penn State College of Medicine, Hershey, Pennsylvania

## Abstract

**Importance:**

The prevalence of annual major depressive disorder (MDD) episodes among adolescents in the United States rose from 8.3% in 2008 to 12.8% in 2016. Despite the US Preventive Services Task Force 2009 endorsement and 2016 reaffirmation of universal adolescent MDD screening in primary care, many adolescents are missed, as more than 60% lack annual preventive health visits and MDD screening remains inconsistent.

**Objective:**

To compare the effectiveness of universal school-based screening for adolescent MDD vs the existing process of targeted screening based on observable behavior.

**Design, Setting, and Participants:**

Screening in High Schools to Identify, Evaluate, and Lower Depression (SHIELD) is a randomized clinical trial that will take place in at least 8 Pennsylvania public high schools among at least 9650 students enrolled in 9th through 12th grade. Students will be randomized by grade to either targeted screening (current process) or universal screening (intervention). Students in the targeted screening arm will complete mandated school health screenings, which do not include an MDD screening. These students will be observed through the academic year for referral to the Student Assistance Program (SAP), required in all Pennsylvania schools. If a student exhibits behavior concerning for MDD raised by any contact (eg, teacher, parent, peer, or self-referral), SAP will triage the student and provide follow-up recommendations. Students in the universal screening arm will complete the validated Patient Health Questionnaire-9 (PHQ-9) from September through December of the academic year. The PHQ-9 includes 9 close-ended questions and is scored from 0 to 27. Students with a positive result (ie, score >10) will proceed to SAP triage. Students in the intervention arm will also be observed for behavior concerning for MDD during the school year, potentially prompting SAP triage referral. The primary outcome will be the proportion of adolescents referred to SAP triage who are recommended for additional MDD-related services and successfully engage with at least 1 SAP recommendation. Observers will not be blinded to patient groups, and an intention-to-treat analysis will be used.

**Discussion:**

The SHIELD trial began with 3 schools during the 2018-2019 academic year. Screening in the intervention arm with the PHQ-9 is currently underway for the remaining schools, with a goal of completion of all PHQ-9 screenings by December 2019. This trial addresses the US Preventive Services Task Force call for large, high-quality randomized clinical trials to better understand the effects of MDD screening and quantify the proportion of adolescents with screen-detected MDD successfully referred and treated.

**Trial Registration:**

ClinicalTrials.gov identifier: NCT03716869

## Key Points

**Question:**

Is universal school-based screening for adolescent major depressive disorder (MDD) more effective than the existing process of targeted screening based on observable behaviors of concern?

**Findings:**

Screening in High Schools to Identify, Evaluate, and Lower Depression (SHIELD) is a randomized clinical trial that will take place in at least 8 public senior high schools in Pennsylvania to evaluate the effectiveness of universal screening for identifying MDD and engaging students with resources.

**Meaning:**

The SHIELD trial directly addresses the US Preventive Services Task Force call for large, high-quality randomized clinical trials to better understand the effects of MDD screening and quantify the proportion of adolescents with screen-detected MDD successfully referred and treated.

## Teaser

This trial protocol describes a randomized clinical trial that will compare the effectiveness of universal school-based screening for adolescent major depressive disorder vs the existing process of targeted screening based on observable behavior.

## Introduction

The prevalence of annual major depressive disorder (MDD) episodes among US adolescents rose from 8.3% in 2008 to 12.8% in 2016.^1^ Approximately 30% of adolescents with MDD report some form of suicidality, with more than 10% making a suicide attempt.^2^ In 2017, suicide was the second leading cause of death among youth aged 10 to 24 years.^3^

Significant increases in MDD episodes have been observed for all racial and ethnic groups except non-Hispanic black individuals, who demonstrated a smaller but still increasing trend.^4^ The most striking increase in MDD episodes has been for women across all racial and ethnic groups.^1,4^

Data from Healthy People 2020, based on the National Ambulatory Medical Care Survey, found that 2.1% of adolescent primary care office visits included MDD screening from 2005 to 2007.^5^ Additionally, a study using National Ambulatory Medical Care Survey data^6^ found primary care adolescent MDD screening was 80% less likely for Hispanic adolescents compared with non-Hispanic white adolescents. Similar inequalities were reported for women, who were 3 times more likely to have MDD but less likely to be treated than men.^1,2^ Despite the US Preventive Services Task Force (USPSTF) 2009 endorsement and 2016 reaffirmation of universal adolescent MDD screening in primary care, MDD screening remains inconsistent.^5,7,8^

We propose that schools may provide an ideal setting to conduct universal MDD screening and compare the effectiveness of universal vs targeted MDD screening.^9,10^ Most adolescents in the United States lack annual preventive health visits.^11,12^ However, most adolescents, regardless of race, ethnicity, and socioeconomic status, attend school. Adolescents’ consistent contact with schools compared with the medical setting has been used to advocate for physical school-based universal health screenings for concerns that affect academic success (eg, vision, hearing).^13^ Adolescent MDD similarly has negative consequences for academic performance.^7,14,15^ While school-based health clinics have been helpful in the management of adolescent behavioral health, only 2000 exist in the United States, and not all high school settings have them.^16^ Schools provide a critical access point to equalize disparities in MDD identification and treatment.

In Pennsylvania public schools, the primary process to address the needs of students who display any barrier to learning is the Student Assistance Program (SAP). The SAP operates in all 500 school districts in Pennsylvania and uses a team process to gather and review data on observable behaviors related to the referral concern and, if appropriate, to refer the student for further screening or assessment by a behavioral health liaison, who can provide referrals to school-based or community-based behavioral health resources.^17,18^ The SAP team composition varies across school districts but typically includes a minimum of 4 trained staff members. Students may self-refer, but most SAP referrals depend on a student exhibiting concerning behavior that is detected by school staff, peers, or parents. As women and racial/ethnic minorities (eg, Hispanic individuals) tend to present with more internalizing mental health symptoms, the current process is likely to lead to increasing health disparities, which may be better identified with a universal screening approach.^19,20,21^ Universal screening may also address other biases in behavior management among racial/ethnic minority students.^22^

There is precedent for schools to address adolescent mental health via universal screening. Columbia University conducted TeenScreen^23^ from 1999 to 2012, which addressed depression, suicide, anxiety, substance use, and general health in a 10-minute questionnaire. The Developmental Pathways Screening Program,^23^ implemented in 2002 in 4 middle schools in the Seattle, Washington, area successfully linked 78% of students with follow-up services.

The current randomized clinical trial (RCT), Screening in High Schools to Identify, Evaluate, and Lower Depression (SHIELD), focuses solely on adolescent MDD screening among senior high school students (ie, grades 9-12). The RCT is composed of 2 distinct yet complementary studies supported by the Health Resources and Services Administration and the Patient-Centered Outcomes Research Institute (PCORI). Partnering with at least 8 public high schools in Pennsylvania, we will randomize schools by grade to compare the effectiveness of universal school-based screening for adolescent MDD vs the existing process of targeted screening through the SAP process. Aims specific to the Health Resources and Services Administration–funded portion are as follows: (1) to examine the impact of universal screening on the number of adolescents with MDD screened, identified, and engaged in treatment; (2) to examine the effectiveness of universal screening on female adolescents; and (3) to examine the effectiveness among students living in rural communities. Complementary aims specific to the PCORI-funded portion are as follows: (1) to examine the effectiveness of universal screening among urban adolescents and (2) to examine the effectiveness among racial/ethnic minority students, specifically urban non-Hispanic black and Hispanic students.

## Methods

### Participants

Students in grades 9 through 12 at a participating school in Pennsylvania will be eligible for enrollment. In advance of screening, letters will be sent to parents of all high school students. The letters will detail the project and include a copy of the screening tool, the Patient Health Questionnaire-9 (PHQ-9).^24^ Parents will be able to opt their student out of the study. Students in the universal screening arm will also have the opportunity to opt out at the time of screening. We planned for a 20% opt-out rate given the experience of similar school-based studies.^25^

### Randomization

**Figure 1.**
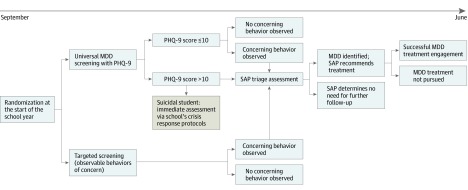
Screening in High Schools to Identify, Evaluate, and Lower Depression (SHIELD), A Randomized Clinical Trial of Universal vs Targeted Screening for Adolescent Major Depressive Disorder (MDD) PHQ-9 indicates Patient Health Questionnaire-9; SAP, Student Assistance Program.

**Figure 2.**
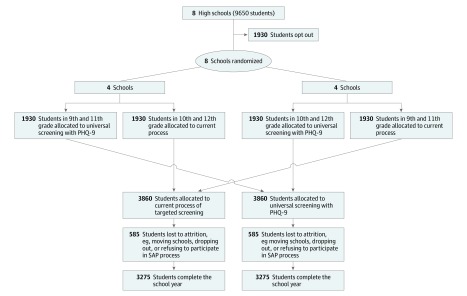
Study Flow Diagram PHQ-9 indicates Patient Health Questionnaire-9; SAP, Student Assistance Program.

Schools will be randomized to 2 groups: (1) 9th and 11th graders assigned to universal screening and (2) 10th and 12th graders assigned to universal screening. The other grades within the school will be assigned to targeted screening (Figure 1 and Figure 2). As the optimal frequency of MDD screening is unknown, those randomized to universal screening will receive the PHQ-9 once between September and December. Students, school staff, and study personnel will not be blinded to randomized group at each school site.

### Control Arm

The targeted screening arm (control arm) will follow the current process. If a student exhibits behavior concerning for MDD raised by any contact (eg, teacher, parent, peer, or self-referral), the SAP team will gather additional data to determine whether a screening or assessment by a behavioral health liaison may be warranted to provide recommendations for school-based or community-based services. As MDD itself is not listed as an incoming referral reason on existing SAP reports, this will be based on a potentially related, primary or secondary MDD-related referral reason.^26^ Students endorsing suicidal thoughts will be managed via the school’s existing crisis response protocols.^27^

### Intervention Arm

The universal screening arm (intervention arm) will have students complete the PHQ-9, which includes 9 close-ended questions and is scored from 0 to 27. Scores higher than 10 are considered a positive result.^24^ The last unscored item, regarding difficulty in function, is not included in the scoring or reported to schools.^28^ Students with a positive result will be referred to SAP using the same process as those in the targeted screening arm. For item 9, which asks about suicidal thoughts, any response other than not at all will be immediately addressed by the school’s existing crisis response protocols.^27^ Throughout the rest of the academic year, students presenting with any behaviors of concern may be referred to SAP at any time via the current process, as described for the targeted screening arm (Table 1).

**Table 1.** Schedule of Enrollment, Interventions, and Assessments

Trial Phase	Study Period				
	Enrollment, June-August	Allocation, July-August	Universal Screening, September-December	Tracking, September-June	Close-out, July-August
Enrollment	X				
Opt-out letters		X	X		
Intervention			X		
Student receives positive screen results			X		
Student identified by observable behaviors of concern				X	
Student triaged by SAP				X	
Student engaged in treatment				X	
Assessment					
SAP reporting				X	X

Abbreviation: SAP, Student Assistance Program.

### Measures

The primary outcome, termed *MDD composite*, will be the percentage of students who meet all of the following criteria: (1) a positive MDD screen by PHQ-9 (universal screening) or referral to SAP for MDD during the school year, (2) SAP triage confirming MDD in need of follow-up (SAP triage is not diagnostic, so this is based on SAP follow-up recommendations for MDD-related school or community services, eg, Mental Health Assessment^26^), and (3) engagement with at least 1 SAP recommended service or treatment for MDD.

Secondary outcomes include the percentage of students who meet the criteria for each component of the primary outcome and the percentage at risk of suicide, which will be measured by a positive response to item 9 on the PHQ-9 or referral to the crisis team for suicidal thoughts, suicide attempts, or completed suicides independent of the screening. Additional secondary outcomes will be collected in aggregate by grade level at each school and include the following: standardized test scores, school policy violations and suspensions, missed school days, grade point average, grade advancement, and graduation rate.

### Sample Size

This RCT is planned in 8 schools with an estimated enrollment of 9650 students. Student populations were estimated from the Pennsylvania School Performance Profile at the time of grant submission.^29^ However, owing to the reality of conducting research in a school setting, the number of participating schools and students may be different than what was planned. Some school districts have experienced leadership changes or have implemented new programs requiring time and resources that could affect their ability to participate. Official enrollment numbers for the school are not available until the fall of the academic year, and some school districts may see their projected enrollment numbers change. At a minimum, we will include 8 schools and 9650 total students as planned in the original submission, although additional schools may be added to the study.

School participation will be staggered across the 2018-2019 and 2019-2020 academic years. We do not anticipate any major changes over the course of 2 academic years that would significantly alter the results compared with conducting the RCT in the same academic year. Staggering enrollment will give the research team the opportunity to troubleshoot any unanticipated issues with the participating schools in the first year.

The study was powered to detect a 3% increase in the primary outcome (ie, MDD composite) for universal screening compared with targeted screening (ie, 5% vs 2%). The 2% rate predicted in the targeted screening group was based on SAP data dating back to 2008, which fluctuated between 1% and 2% of students.^17^ Data from the 2015-2016 school year showed 1.2% of students in Pennsylvania had an SAP referral related to mental health or substance abuse, with 75% of those students further engaging with treatment.^17,30^ The 5% rate in the universal screening group was based on an estimated 12.5% prevalence of adolescent MDD in 2015.^1^ We used the lower rate of 5% because of potential self-selection bias for those who opt out and inclusion of treatment engagement in the primary outcome. Additionally, an increase from 2% to 5% was considered clinically relevant. Sample size estimates were based on an estimated total enrollment of 9650 students. The overall rate of parental and student opt-out is expected to be approximately 20%, leaving approximately 7720 students eligible to be enrolled for the study. Assuming a 15% attrition rate among those enrolled (eg, students who move or drop out of school), the study is expected to include at least 6550 students with complete data (Figure 2). A total of 3275 students in each randomized group yields power greater than 99% to detect a difference of 2% vs 5% using a 2-sided test conducted at a type I error rate of 5% in a mixed-effects logistic regression model. These calculations were based on MLPowSim, a software program provided by the Centre for Multilevel Modeling at the University of Bristol to determine power for mixed-effects models.^31^ For the mixed-effects logistic regression model used to determine power, the difference between study groups corresponds to an intercept parameter of −3.48 and a study group parameter (universal screening vs targeted screening) of 0.73. The parameter for the random effect of schools was assumed to be normally distributed, with a variance specified to be 1, which corresponds to an intracluster correlation coefficient of approximately 0.25. The targeted sample size also allowed for adequate power for secondary analyses.

### Statistical Analysis

The principles of intention to treat will be used for all statistical analyses related to primary and secondary aims. For the primary aim of comparing universal to targeted screening, the statistical analysis will be conducted using a mixed-effects logistic regression model.^32^ The outcome will be an indicator of whether a student met the MDD composite criteria. The model will include a fixed effect for randomized group and a random effect for school. The random effect accounts for correlation among students enrolled within the same school. The primary parameter of interest will be the log odds of the randomized group parameter. Statistical significance of that parameter will be assessed using a 2-sided Wald test, with statistical significance set at *P* < .05. Point estimates for the odds ratio and corresponding 95% CIs will be reported.

We plan to conduct 3 separate subgroup analyses to evaluate whether universal screening is effective compared with targeted screening in lessening the disparities in identifying and treating MDD in different subgroups. The 3 planned subgroups are race/ethnicity (ie, non-Hispanic white; non-Hispanic black; Hispanic, any race; and other race/ethnicity groups), sex (ie, male and female), and school location (ie, urban and rural). For these planned subgroup analyses, the same mixed-effects logistic regression modeling framework will be used, but the model will be extended by including a fixed effect for subgroup and an interaction effect for subgroup by randomized group. The interaction terms will be the parameters of interest. The subgroup analyses for race/ethnicity and sex are considered hypothesis driven (ie, confirmatory), whereas the subgroup analysis for school location will be considered hypothesis generating.

The subgroup analyses for race/ethnicity and sex were adequately powered given our planned sample size. These power calculations were based on expected percentages of MDD treatment engagement by subgroup and randomized group, as shown in Table 2. These expected percentages were determined using data from Healthy People 2020.^1^ For the power calculation of the subgroup analysis for race/ethnicity and in accordance with the most recent data for the 8 schools to be included in the study, we assumed that 34% of students were non-Hispanic white, 13% of students were non-Hispanic black, 50% of were Hispanic of any race, and 3% identified as other races/ethnicities. The power to detect significant interaction parameters in the model for non-Hispanic black students and Hispanic students of any race (with non-Hispanic white students as the reference group) was 86% and 87%, respectively. For the power calculation of the subgroup analysis of sex, we assumed 52% of students were male and 48% of students were female. The power to detect a significant interaction effect was 91%. These calculations were based on the same model specifications, variance components, and software as in the primary analysis.

**Table 2.** Expected Percentages of Adolescents Identified by Universal or Targeted Screening and Engaged in Recommended Services or Treatments by Subgroup

Subgroup	%^a^	
	Targeted	Universal
Race/ethnicity		
Non-Hispanic white	2	5
Non-Hispanic black	1	5
Hispanic, any race	1	5
Other	1	5
Sex		
Male	3	5
Female	2	6

^a^ Indicates students referred to the Student Assistance Program with concerns for major depressive disorder (either through the universal or targeted screening study arm), who are recommended for additional follow-up and successfully engage in a recommended service or treatment.

Other secondary outcomes will be collected in aggregate at the grade level for each school. We will have only 32 data points for these secondary outcomes (4 grades in 8 schools). Mixed-effects linear and logistic models will be used, as appropriate, with a fixed effect for randomized group and a random effect for school. These analyses will be considered hypothesis generating.

Efforts will be made to ensure completeness of data where possible, but missing data will likely occur for a number of anticipated reasons. First, a student may move during the school year to another school district or drop out of school entirely. Second, parents or students may opt out at any time during the school year. Third, students in the universal screening arm may leave the PHQ-9 incomplete. We have planned for a large rate of attrition (ie, 15%) because of uncertainty regarding the numbers of students who may have missing data because of these anticipated and unanticipated reasons. For students with missing data, the outcome variable will be unknown and considered missing. We expect that missing data will occur completely at random. The mixed-effects models proposed for the primary and secondary analyses are based on a maximum likelihood estimation, which leads to valid and unbiased conclusions in the presence of data missing completely at random.^33^ Multiple imputation will be used for the subgroup analyses when missing demographic variables occur. The method has been shown to yield estimates comparable with those obtained if all data were present under the assumption of missing at random.^34^

### Data Collection

To ensure confidentiality, enrolled students will be assigned a study identification (ID) number. The school will maintain a master list tying study ID number to identifiable student information, and the study team will only have access to deidentified data. Adolescent participants in the targeted screening arm will not have increased risk of loss of confidentiality because current school policy will be used. For those in the universal screening arm, the PHQ-9 screening will be administered on an iPad (Apple, Inc) with an internet connection, which allows direct entry of the results into Research Electronic Data Capture (REDCap), a secure, web-based application designed to support research studies.^35^ The PHQ-9 is available in multiple languages, and based on discussion with participating schools, it will be offered to students in either English or Spanish.^28^ After an individual student has completed the PHQ-9, the research assistant will take the iPad, refresh the page, and reopen the next survey using the assigned student study ID number. This process ensures that subsequent participants have no access to prior responses by their peers and that no identifying information leaves the school building. To immediately identify a student indicating suicidal intent, the survey will flag positive responses to question 9 in real time. The research assistant will relay the study ID number of the student of concern to preidentified school staff.

Students with elevated PHQ-9 scores will also be shared via study ID number for follow-up through the school SAP team. Any subsequent referrals to the school SAP team from either the intervention or control arm will be tracked. This tracking is part of the existing school process as well as the annual SAP report to the state department of education.^26^ The information collected by the study team at the end of the year will be the school’s state SAP report at the individual level by study ID number and include no identifying information. Demographic characteristics will include age (in years), sex, and race/ethnicity per school records for each student. Sex will be restricted to male and female as this is how the current school data reporting form is configured.^26^

### Institutional Review Board

The RCT was approved by the Penn State Institutional Review Board on August 27, 2018. This report follows the Standard Protocol Items: Recommendations for Interventional Trials (SPIRIT) reporting guideline. The full trial protocol is available in the Supplement.

### Data and Safety Monitoring Board

A data and safety monitoring board will meet twice during the 3-year study period. While completion of the PHQ-9 is low risk, mental health and depression are often a difficult and controversial topic for communities. The data and safety monitoring board may provide valuable input and oversight related to school and community challenges that arise in the course of the study.

### Qualitative Components and Community Engagement

Support from PCORI requires the formation of a stakeholder advisory board with representatives that include school staff, parents, students, SAP regional coordinators, and suicide-prevention organizations, all of whom have provided guidance and feedback for the initial design phase of the study. The group will provide ongoing guidance for the RCT as well as inform the dissemination of study findings. The PCORI protocol includes a qualitative component intended to engage with individual school communities around the topic of adolescent MDD. Prior to the RCT, focus groups of students and parents (each consisting of approximately 10-12 participants) will be run separately at schools supported by PCORI. The goal of these focus groups is to identify barriers to MDD screening and treatment within individual communities. Similarly, interviews will be conducted with 2 to 3 staff members at each of these schools to identify and address barriers to the screening process. Following the RCT, interviews will be conducted with students, parents, and staff at each school regarding their opinions of the screening process, concerns, and thoughts on sustainability.

Additionally, all schools will have the opportunity to engage in community events to promote what the school is doing and foster the discussion around student mental health. Numerous options have been proposed with school input. For example, funds may support student mental health club activities, a movie screening with panel discussion, or a book club and facilitated discussion. Funds have also been allocated for school staff to participate in the Question, Persuade, Refer Gatekeeper Training for Suicide Prevention targeted to school health professionals.^36,37^

## Discussion

Our proposal is uniquely positioned to inform the current evidence regarding adolescent MDD screening. First, our proposal does not attempt to establish universal screening for multiple mental health issues but focuses solely on the USPSTF recommendation for universal adolescent MDD screening.^8^ Second, the study uses a validated, USPSTF-endorsed screening questionnaire, the PHQ-9, which is readily available and free of charge online to schools and is published in multiple languages.^7,8,24,28^ Third, the process following a positive PHQ-9 screen uses existing school resources, specifically SAP. The research team will partner with school SAP teams prior to screening implementation to collaboratively map current mental health resources, identify new resources, and plan an approach to addressing positive results in the universal screening group.

### Limitations

This study has limitations. The primary study outcome includes treatment engagement, which is not a substitute for completion but a realistic proxy. As SHIELD is a pragmatic clinical trial, the study cannot be conducted in an ideally controlled setting. However, it is also anticipated that this approach will make the findings more broadly and practically applicable to the community.

## Conclusions

The SHIELD trial addresses the USPSTF call for large, high-quality RCTs to better understand the effects of MDD screening and quantify the proportion of adolescents with screen-detected MDD successfully referred and treated. While the hope is that universal screening will be beneficial for adolescents and provide a means to address the current epidemic of adolescent MDD and suicide, the RCT results may not support this outcome at multiple levels. At a minimum, by engaging schools and communities and fostering dialogue on adolescent depression, we may further reduce the stigma surrounding MDD and formally evaluate means to improve the critical first step in a pathway that has proved persistently challenging to address.

## Notes

Supplement. **Trial Protocol**
